# Dr. Talbot and His Experiments with Mercury, Amalgams, Etc.

**Published:** 1882-04

**Authors:** Chas. Mayr


THE
AMERICAN JOURNAL
OF
DENTAL SCIENCE.

Vol. XV. THIRD SERIES—APRIL, 1882. No. 12.

### ARTICLE I.

### *Dr. Talbot and his Experiments with Mercury, Amalgams, Etc.*

BY CHAS. MAYR, A. M.

Already in the February number of the *Journal* we propose to enter upon some of Dr. Talbot's remarkable conclusions, etc., in his paper: On the Effects of Mercury in Amalgam Fillings, as published in the *Ohio State Journal*, January, 1881. The paper first gives a very good synopsis of the grossly chemical facts about mercury. Thus far we agree with the author. But there comes a "Wort zur rechten Zeit!" He says: "The better class of dentists waged war against amalgam on general principles, not alone on account of the deleterious effects of the mercury in its composition, but because of its unsightly appearance and demoralizing effects upon the dental profession." Let us never lose sight of the necessity to separate completely "demoralizing effects upon the profession," and "deleterious effects of the mercury." Let us be honest, and consider why the best practitioners think amalgams demoralizing and are then easily persuaded into their harmfulness. We need not enter into details; every dentist understands the point of "demoralizing," but let not *truth* get demoralized together with the pocket-book. Each point has to be settled alone without reference to the other. We grant to the author the unsightly appearance of amalgams—where they are seen; we admit to him their "demoralizing effect," though we might rather call it "depressing effect;" we think this quite a good and legitimate ground upon which to fight amalgams; but his assertions about the physiological effects of the mercury in amalgams we have to declare as chemical and physiological superstitions.

We notice with pleasure the scientific statement: "The rapidity with which the evaporation of mercury takes place depends upon the amount of heat and the surface exposed, and not upon the quantity of mercury in the filling." This is perfectly correct. Another point to be mentioned as correct is, that after three months' standing no weighable amount of mercury had evaporated from an amalgam. Dr. Talbot only confirms still more an old observation. His experiments again are quite correct, and no flaw can be found in these fundamental facts; it is well known that mercury vapor is strongly condensed on gold, and one might even object to this rapid condensation as creating unnatural conditions. But, as we will have to show that the experiments had less to do with the amalgam question than with a new process of making black designs on paper, we will not pick up trifling objects here. If Dr. Talbot had left off the rest of his paper after having related his experiments, it might have been a good lecture; but, by trying to draw conclusions from these experiments, he proves that reasoning soundly and making good experiments are two entirely different things. Dr. Talbot is—speaking scientifically—a good stone gatherer, but as soon as he tries to make a building with these stones he gets nothing but a shapeless heap.

His assertion: "I am in possession of numberless cases of poisoning from mercury in amalgam-fillings," in this general dogmatic form, can be answered only by one thing: "Doctor your fancy is very powerful!" He reports a case from the *Dental Register*, 1872, based solely on the saying of two doctors. If *they* agreed that "he was suffering from the effects of mercury, present in the amalgam used in filling one of his teeth," does that prove the very least? The whole story of the doctors and the jury reads so "popular," so utterly unscientific and worthless, that we wonder how Dr. Talbot for a moment could take stock in such a vague statement, if it was not because it suited his idea. We would believe the story to be correct, if at least several hundred thousands of deaths or severe mouth diseases of the same character were reported. But does the doctor really think because an ignorant jury ascribes an œdema glottidis to mercury in amalgams or to the man in the moon, that this proves much for either case?

Just as worthless as such *isolated*, more than doubtful, cases are quotations from authors without sufficient reason to apply them to the point under discussion. No doubt Dr. Bartholow is all right in ascribing to mercury as inhaled, "wasting, ptyalism, etc.," but he speaks of gilders and others, hence of people who inhale quantities, many thousand and thousand times more than can ever evaporate from an amalgam, which as Dr. Talbot says did not lose a "weighable amount of mercury in three months." Just the quoted case from Walter Pope, of a workman who had not handled mercury for six months and yet rendered a piece of copper as white as silver, if true, proves that enormous quantities of mercury may be stored up in the body—as most experimenters agree, in the shape of metallic globules—without killing the man. Quite perplexing reads the statement:

"Is it not a reasonable supposition that, if poisonous symptoms are produced in proportion with the subdivision of the particles of mercury, that the system will be more severely affected by the vapor of mercury, which is finer than any mechanical subdivision?" Of course, doctor, of course! What we have to dread are not so much those six-grain doses of mercury, but those *one* quintillions of grains; they kill! And, further, applying the same sound (?) logic to our dangerous amalgams, the smaller the amalgam the more finely subdivided the vapors from it by being mixed with more air, hence the more dangerous and by simple consequence—doctor, we do not joke!—the amalgam, most appropriating nothing, will give off the highest dilution of vapor, hence be most powerful and dangerous; therefore, chiefly those cases where the amalgam plugs have very small surfaces will be dangerous—granting for one moment Dr. Talbot's supposition about those mercurial vapors!

This great danger from those microscopically small amalgams in comparison with the larger ones, as would follow from the basis of Dr. Talbot, receives a kind of support by the story of the crew of the "Triumph," where in spite of the mercury coating all the metal about the ship, only "nearly all the crew were salivated." Surely, if only one-mililionth of a grain had evaporated they all would have died! We only wish to prove ad absurdum a most fanciful, hypothetical, inconsequent way of thinking, as the one is that, the higher the peculiar trituration of homœopathic drugs, the better the effect. The bottomless hypothesis, dating from a time when all about chemistry and physiology was ignorance, unfortunately still now and then takes hold of people of fanciful dispositions. We do not wish to be understood that we declare all homœopathy erroneous; there is a certain limit for all drugs upward and downward. The man who prescribes above the physiologically required limit, is an unscientific, dangerous quack; the man who prescribes so far below the exactly required quantity that it has no effect, is an unscientific, superstitious enthusiast, who may be dangerous by neglect. Practically, it is true, if we have to choose between either of the extremes, we rather wish to die naturally without drugs than be killed by drugs, hence we would rather have a "homœopath" than an "allopathic" quack; but scientifically both extremes are equally wrong; the really scientific doctor is in the position of the painter who asked what he mixed with his colors to give such fine effects. "Brains!" was the answer. This is the most valuable drug known, but the friends of the ℞. will not always find it among the prescriptions, be it of a "homœopath" or "allopath."

But let us once more turn to Dr. Talbot. He makes experiments on roaches and mercury and amalgams. Though we have to feel sorry that a high dilution of mercury-vapor destroyed their scientific thirst, yet we would suggest to repeat the experiments, enclosing the roaches *with some food* in the bottle, lest wicked people might say we starved them, while we tried to kill them by mercury. How accidental some of those death-rates are, is shown by the fact that in the same bottle one roach outlived the other by seven days. Those experiments prove nothing even thus. A roach is brougut in close proximity to a large quantity of mercury; a roach weighing probably not five grains is surrounded by ten grains of mercury. As the doctor applies his experimeus to amalgam-filling, we have to suppose that a weight of mercury of twice the weight of the body is evaporating in the mouth or around the body. In order to have such experiments worth anything, the experimenter must possess a slight idea of figure proportions. To bring a roach in the atmosphere of twice its weight of mercury, and to compare the result with the effect of an amalgam weighing one seventy-thousandth of the human body reminds us of the boy who got struck by an engine and knocked over, and in afterwards essaying on the properties of Iron, wrote: it is used for needles, it knocks people down and is used for steel pens. About the effects of mercury in amalgams on plants, a great number of experiments have been made by Runge, with pretty nearly opposite results from those of Dr. Talbot. His experiments four and five are of the same inclusive kind; in one case a guinea pig is put in a four-quart glass jar with *four ounces* of mercury ! ! When we investigate the effect of *amalgams* we ought not to surround the animal with twice its weight of *pure mercury.* For one cockroach we have to give about—say liberally !—1–2000th of a grain, but not 20,000 times as much ! ! Now to that vapor of mercury First, Regnault has done vastly more than Dr. Talbot ; he has measured the exact tension of mercury-vapor for every degree of temperature. At 0° C. it is .027 millimeters, etc. Regnault has further proved that at 60° Fahrenheit one liter (one quart) of air contains about one-half milligram,—one one-hundredth of a grain—of mercury-vapor *if saturated* ; now as Dr. Talbot himself acknowledges that in three months the amalgams did not lose weighable amounts, there follows, that in three months a person would not inhale one quart of mercury, having a degree of rarefaction as the air has in about two hundred miles' height. If the doctor wishes to prove anything about amalgams with his roaches, the only thing corresponding would be either to put his roaches into a large box, with plenty of food, air and exercise, and introduce into the box for every roach one two-thousandth of a grain of *amalgam;* better still to insert one two-thousandth of a grain of amalgam into the mandibles or tongues of those creatures, and if they die they prove nothing. Also here the experiments with the roaches lead to nothing but worthless conclusions. Dr. Talbot will hardly have fancy enough to deny the fact that millions of people wear "tons of amalgams," and live as happy with as healthy mouths as other millions who have gold cropping out at their teeth.

In the discussion which followed the paper, there showed itself an appalling amount of homœopathic superstition. Dr. Cushing alone did not follow the drift of the day but put the thumb on the sore spot of the whole paper, that the blackening of slips, the killing of roaches, guinea pigs and dogs did not prove anything for or against amalgams.

Dr. Spalding made a statement of scraping mercury from the mucous membrane of the mouth, so that globules could be seen. In a paper we reprint in this number, Dr. Spalding shows too much common sense to be supposed to exaggerate about the above mercury-globulus ; yet if it proves anything, it proves that even immense quantities of mercury may be tolerated in the system without the effects attributed by Dr. Talbot to a single amalgam plug!

Dr. Taft.—Without doubt a very honest skeptic—never used amalgam, and he, of course, hails with joy everything supporting his dislike. But dislikes are nothing scientific. He dreads the vapor of mercury. But let us follow a little this vapor from the plugs, as the vapor has formed at the temperature of the mouth, it can only become condensed—as globules—at temperatures *lower* than that of the mouth. But the lungs have a higher temperature, hence no condensation takes place there, but nowhere else in the body does the vapor find any place for condensing; nay, even granting for a moment, that the vapor like that of oxygen may become absorbed by the blood, the surface of the whole body will evaporate the mercury absorbed and, as Dr. Talbot himself admits, in proportion to the surface. Now supposing an amalgam-filling of only (!) one square inch area, the area of the body being about 2,000 square inches, there will mercury evaporate into the surrounding air 2,000 times faster than it is taken up; hence to talk about cumulative effects under such conditions would be sheer nonsense. Besides, we must never forget that while man breathes about 27,000 cub. ft. of air in three months, the quantity of mercury evaporatng in that time could not be weighed by Dr. Talbot himself. Yet granting that one-half milligram evaporated, this was diluted with 9,477 million times its weight of air; it was furthermore—if possible—secreted 2,000 times faster than inhaled, and yet those roaches died from the vapor of twice their weight of pure mercury, and lots of people die every year from—railroad collisions. "Thy faith hath made thee whole." If these are the kind of experiments upon which homœopathy rests—and the drift of the discussion shows that they were accepted by the homœopaths in the meeting as proving something—we need not wonder that *scientific* (?) homœopathy has become such a monstrous absurdity; and as there will always be plenty of people unable to distinguish between "post hoc" and "proptee hoc," between an "after it" and "because of it," homœopathy will always have followers who find causal connections between "carbo" and a ceasing tooth ache! We should like very much to get some more such conclusive experiments from Dr. Talbot; but let there be at least an approximate or distant connection between the experiments and the theory which is to be proved by them.—*New England Journal of Dentistry.*

